# No inequalities in survival from colorectal cancer by education and socioeconomic deprivation - a population-based study in the North Region of Portugal, 2000-2002

**DOI:** 10.1186/s12885-016-2639-9

**Published:** 2016-08-05

**Authors:** Luís Antunes, Denisa Mendonça, Maria José Bento, Bernard Rachet

**Affiliations:** 1 Department of Epidemiology, Portuguese Oncology Institute (IPO Porto), Porto, Portugal; 2 RORENO - North Region Cancer Registry of Portugal, Porto, Portugal; 3 Faculty of Sciences, University of Porto, Porto, Portugal; 4 EPIUnit – Institute of Public Health – University of Porto (ISPUP), Porto, Portugal; 5 Institute of Biomedical Sciences Abel Salazar, University of Porto, Porto, Portugal; 6 UMIB, Institute of Biomedical Sciences Abel Salazar, University of Porto, Porto, Portugal; 7 Cancer Survival Group, London School of Hygiene and Tropical Medicine, Keppel Street, London, WC1E 7HT UK

**Keywords:** Net survival, Colorectal cancer, Education, Deprivation, Inequalities, Life tables

## Abstract

**Background:**

Association between cancer survival and socioeconomic status has been reported in various countries but it has never been studied in Portugal. We aimed here to study the role of education and socioeconomic deprivation level on survival from colorectal cancer in the North Region of Portugal using a population-based cancer registry dataset.

**Methods:**

We analysed a cohort of patients aged 15–84 years, diagnosed with a colorectal cancer in the North Region of Portugal between 2000 and 2002. Education and socioeconomic deprivation level was assigned to each patient based on their area of residence. We measured socioeconomic deprivation using the recently developed European Deprivation Index. Net survival was estimated using Pohar-Perme estimator and age-adjusted excess hazard ratios were estimated using parametric flexible models. Since no deprivation-specific life tables were available, we performed a sensitivity analysis to test the robustness of the results to life tables adjusted for education and socioeconomic deprivation level.

**Results:**

A total of 4,105 cases were included in the analysis. In male patients (56.3 %), a pattern of worse 5- and 10-year net survival in the less educated (survival gap between extreme education groups: -7 % and -10 % at 5 and 10 years, respectively) and more deprived groups (survival gap between extreme EDI groups: -5 % both at 5 and 10 years) was observed when using general life tables. No such clear pattern was found among female patients. In both sexes, when likely differences in background mortality by education or deprivation were accounted for in the sensitivity analysis, any differences in net survival between education or deprivation groups vanished.

**Conclusions:**

Our study shows that observed differences in survival by education and EDI level are most likely attributable to inequalities in background survival. Also, it confirms the importance of using the relevant life tables and of performing sensitivity analysis when evaluating socioeconomic inequalities in cancer survival. Comparison studies of different healthcare systems organization should be performed to better understand its influence on cancer survival inequalities.

**Electronic supplementary material:**

The online version of this article (doi:10.1186/s12885-016-2639-9) contains supplementary material, which is available to authorized users.

## Background

Colorectum is the second most common cancer site in the North Region of Portugal, only surpassed by prostate in men and breast in women [1]. Age-standardized incidence rates of both colon and rectal cancers have been recently rising in this region of Europe and are predicted to continue rising, at least until 2020 [2, 3]. Five-year survival from colorectal cancer (CRC) in Portugal was generally higher than in Eastern European countries, the UK, Denmark and Spain, and lower than in The Netherlands, France, Italy and the Nordic countries among others [4].

Association between survival from colon or rectal cancer and socioeconomic status (SES) has been repeatedly reported in various countries [5, 6]. Socioeconomic condition can be attributed to each patient using individual measures [7–9]. However, population-based cancer registries rarely collect individual data on socioeconomic factors. Alternatively, ecological (area-based) measures are used [10–12]. Although not reflecting the individual condition of each patient, ecological measures are informative enough to evaluate the association between SES and survival from cancer, as long as the population size of the areas considered is sufficiently small and homogeneous relatively to the SES measure [13]. The SES can be measured using single indicators (e.g., income, education) [9, 14] or composite indices (e.g., Townsend, Indices of Multiple Deprivation) [10, 11, 15]. Because the large number of different indicators found in the literature can hamper comparisons between studies, a new ecological socioeconomic deprivation index (European Deprivation Index – EDI) has been recently developed for several European countries (Portugal, Spain, France, Italy, England), based on the same methodology across all countries [16]. The index is derived from country-specific census variables that are most associated with the variables of the survey European Union-Statistics on Income and Living Conditions EU-SILC [17].

Independently of the SES measure, patients with a lower SES are generally found to present a worse survival compared to patients with a higher SES. Potential reported causes for SES inequalities in survival include variations in stage of disease at presentation, type of treatment delivered or patient characteristics [6, 18].

The National Health Service (SNS) functions in Portugal since 1979 and aims to provide the population with complete and high-quality care, independently of their social or economic condition. Cancer patients were totally exempt of paying moderating fees until the end of 2011. In an evaluation of the Portuguese situation regarding CRC, Pinto and colleagues suggested that one of the major problems in the management of the diagnosis and treatment of colorectal cancer patients were regional disparities in access to health [19]. However, to the best of our knowledge, socioeconomic inequalities in cancer survival in Portugal have not been assessed yet.

In the present study we aimed at evaluating the association between up-to-10-year survival from colorectal cancer and two indicators: the recently developed area-based socioeconomic indicator EDI and education level based on census information. We used population-based data from the North Region Cancer Registry of Portugal (RORENO).

## Methods

### Cancer registry

Cancer data were provided by RORENO, a population-based cancer registry established in 1988. The analyses were performed according to RORENO guidelines ensuring the anonymity of the information used. Its catchment area corresponds to the North Region of Portugal, with 3.2 million inhabitants (around 30 % of the national population). All incident cancer cases occurring in the area were recorded by the registry either directly from the main public hospitals through a web-based platform, or based on the hard copies of the medical reports for the private hospitals and pathology laboratories. Registration quality follows IARC rules [20].

### Data

We considered for analysis all malignant, invasive tumours of the colon and rectum (ICD-10 [21] codes C18-20) diagnosed in adults resident in the North Region of Portugal in the period 2000 to 2002. For patients diagnosed with more than one tumour during the study period, only the first primary tumour contributed to the analysis. Follow-up of each patient was both active (by contacting the institutions where the patient was diagnosed and/or treated) and, when necessary, passive (by obtaining the vital status from the National Health Service database or the Civil Registration Offices). The end of follow-up was 31^st^ December 2012, allowing over 10 years of potential follow-up for all patients. Because 10-year net survival is meaningless for very old patients, the study was restricted to patients aged 15 to 84 years.

### Education and EDI level

No information on education or other SES indicator at individual level is systematically registered by cancer registries in Portugal. Education level and the socioeconomic deprivation index (EDI) were assigned to each patient based on their census area of residence at diagnosis. When not available, patient’s address was completed using the National Health Service database. The residence of each patient was geocoded using a web-based service [22] and then confirmed manually. The coordinates of each patient’s address were then matched with the relevant census area using a Geographical Information System (Arc GIS 10.2).

Education level was measured as the proportion of inhabitants in each census area aged 15 years or plus with at least 9 years of education (compulsory level of education in Portugal until 2009). This information was retrieved from the 2001 national census and the census area (in Portuguese: *secção estatística*) corresponds to the area of a census taker [23] (median population size: 665; range: 13 – 3123; number of sections: 4651). Education level was then categorized in five levels according to the quintiles of the regional distribution of all area-level education proportions. The distribution was weighted by the population size in each census area so that each level corresponds to 20 % of the total population (and not to 20 % of the number of sections). The first category corresponds to the census areas with the lowest proportion of residents with at least the compulsory level of education (proportion lower than 18.0 %) and the fifth category to areas with the highest proportion (proportion equal or higher than 48.9 %). The EDI was attributed to the census areas and categorized in five groups from q1 (the most deprived) to q5 (the least deprived).

### Statistical analysis

Age distribution between groups was compared using Kruskall-Wallis or Mann–Whitney non parametric tests, as applicable. Survival time was considered as the time between diagnosis and death from any cause or end of study period, whichever occurred first. Up-to-10-year net survival was estimated using the Pohar-Perme non-parametric estimator [24]. Net survival is the survival that would be observed if cancer was the only possible cause of death and can be interpreted as the survival from the cancer. To this purpose, it accounts for the other causes of death or expected mortality. Within the relative survival setting, i.e., when the individual cause of death is not reliably known, the background or expected mortality is provided by life tables for the general population, here of the North Region of Portugal. The tables were built by the Cancer Survival Group (London School of Hygiene and Tropical Medicine) for the CONCORD-2 programme [25], using a multivariable flexible Poisson model [26]. The population and death counts to derive the life tables were provided by the national statistics office (Statistics Portugal). Life tables were stratified by sex, single year of age and calendar year.

Excess (i.e., cancer-related) hazards of death are also of interest. Univariable excess hazard models were used to test significance of potential prognostic variables (sex, age group, cancer site). Multivariable flexible parametric models [27] were used to estimate the hazard ratios of excess mortality for education and EDI levels, adjusted for potential confounders. Men and women were analysed separately. Education level and deprivation were kept in the model as categorical variables. Different models for the effect of age on the excess hazard were tested, considering age as categorical or continuous variable, with possible non-linear effect using restricted cubic splines. Time-dependent effects for age, education and EDI level were tested. The model with the lowest Akaike Information Criterion (AIC) was chosen.

All analyses were performed using STATA commands *stns* [28] and *stpm2* [29]. Results were considered statistically significant for *p*-value < 0.05.

### Sensitivity analysis

Socioeconomic condition can affect the mortality of a cancer patient from both their cancer and other causes. Assessing socioeconomic inequalities in cancer survival should therefore account for socioeconomic differences in mortality from other causes (the expected or background mortality) [30]. Ignoring such differences leads to over-estimate the inequalities in cancer survival. Since no education-specific neither EDI-specific life tables are available in Portugal, we performed a sensitivity analysis to test the robustness of the results to the choice of the life tables. We built a series of hypothetical SES-specific life tables for Portugal according to various scenarios of inequalities in background mortality. Under the worst case scenario, we mimicked the wide gap in background mortality observed between socioeconomic categories in England, as illustrated by the English (2001) deprivation-specific life tables (http://csg.lshtm.ac.uk/). We further refer to this scenario as S5, and the scenario with no gap as S0. The worst case scenario (S5) corresponded to a difference in life expectancy between extreme groups of 7.7 years in men and of 4.1 years in women. In scenario S4, we reduced the difference in background mortality between SES groups by 20 %, obtaining a gap in life expectancy of 6.2 and 3.3 years in men and women, respectively. We continue reducing the gap in 20 % steps to produce the other life table sets (S3, S2, S1 with corresponding differences in life expectancy at 4.6, 3.1 and 1.5 years in men, and 2.5, 1.7 and 0.8 years in women) until the gap vanishes (S0). We then re-ran the survival analysis using each of these life tables.

## Results

We identified 4,243 cases of colorectal cancer eligible for analysis over the period 2000-2002. After excluding cases with missing information on their vital status at the end of follow-up (*n* = 113) or on their residence address (*n* = 25), 4,105 cases (96.7 %) were included in the analysis (Table 1). More than half (56.3 %) were male. Distribution of age at diagnosis was similar in both sexes (median 68 years, interquartile range 59-74). Colon cancer patients represented nearly two thirds of the cases (64.0 %) and were slightly older than rectal cancer patients (median 68 versus 67 years, *p*-value = 0.002). The proportion of colorectal cancer patients increased towards the more educated groups. The distribution of patients by EDI level was in the opposite direction, with a higher proportion in the more deprived groups. Median age ranged from 67 to 68 years in the highest and least educated groups (*p*-value = 0.176), and from 66 to 68 years between the least and most EDI deprived groups (*p*-value = 0.056).

**Table 1:** Description of the cases included in the analysis stratified by sex

Variable	Male		Female	
	*n*	%	*n*	%
All	2310	100	1795	100
Age group				
15–44	114	4.9	125	7.0
45–54	268	11.6	209	11.6
55–64	548	23.7	364	20.3
65–74	876	37.9	616	34.3
75–84	504	21.8	481	26.8
Education level				
Higher education	516	22.3	434	24.2
q4	543	23.5	422	23.5
q3	475	20.6	366	20.4
q2	400	17.3	328	18.3
Lower education	376	16.3	245	13.6
EDI				
Least deprived	377	16.3	289	16.1
q4	403	17.4	310	17.3
q3	459	19.9	334	18.6
q2	490	21.2	393	21.9
Most deprived	581	25.2	469	26.1
Cancer site				
Colon	1421	61.5	1206	67.2
Rectum	889	38.5	589	32.8

Net survival at 1, 5 and 10 years since diagnosis was 81.5 % (95%CI: 80.3–82.8), 57.5 % (95%CI: 55.7–59.3) and 51.6 % (95%CI: 49.4–53.8), respectively. No significant differences in net survival were found by sex (*p*-value = 0.460) or cancer site (*p*-value = 0.209). Net survival was significantly lower in elderly patients (aged 75-84 years) than in the youngest age group (*p*-value < 0.001) while no significant differences were found among all other age groups. This pattern was similar in both genders and for both cancer sites (data not shown).

**Fig. 1:**
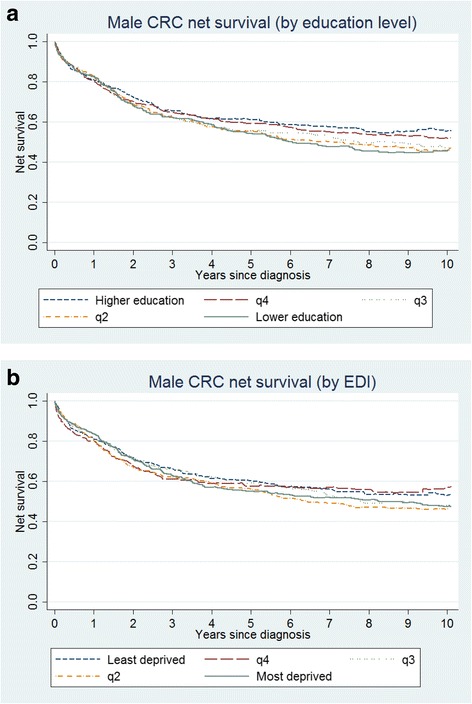
Net survival for male patients: **a** by group of education level and **b** by EDI group (general Life Tables)

For male patients, 1-year net survival estimated using general life tables was similar across education categories, ranging from 80 % to 83 % (Table 2). However, there was an education-related pattern for longer-term survival. The gap in 5- and 10-year survival widened (Fig. 1a), with differences between the two extreme education groups at 7 % and 10 %, respectively. The gradient in net survival by EDI category was not as clear as by education quintile (Fig. 1b). Nevertheless, male patients coming from the least deprived group presented at 5 and 10 years a better net survival than patients coming from the most deprived groups.

**Table 2:** Net survival by education and EDI level at 1, 5 and 10 years after diagnosis^a^

		Male						Female					
		1-year		5-years		10-years		1-year		5-years		10-years	
		%	95 % CI	%	95 % CI	%	95 % CI	%	95 % CI	%	95 % CI	%	95 % CI
Education level													
	Higher education	81	77 – 85	61	56 – 66	56	49 – 62	82	78 – 85	59	53 – 64	57	50 – 63
	q4	80	77 – 84	59	54 – 64	52	46 – 58	83	79 – 86	54	49 – 60	50	44 – 56
	q3	82	78 – 86	56	50 – 61	47	40 – 54	83	79 – 87	57	51 – 63	55	48 – 61
	q2	82	78 – 86	55	50 – 61	46	39 – 53	85	80 – 89	65	59 – 71	56	48 – 63
	Lower education	83	79 – 87	54	48 – 60	46	39 – 53	73	68 – 79	51	44 – 58	46	38 – 54
EDI													
	Least deprived	81	77 – 85	60	54 – 66	53	46 – 60	83	78 – 88	60	54 – 67	58	50 – 65
	q4	80	76 – 84	58	52 – 64	57	49 – 64	79	74 – 83	60	53 - 66	53	46 – 60
	q3	82	78 – 85	59	54 – 65	48	41 – 54	81	77 – 86	56	50 – 62	49	42 – 56
	q2	80	77 – 84	56	51 – 62	46	40 – 53	84	80 – 88	56	50 – 61	52	45 – 58
	Most deprived	84	80 – 87	55	50 – 60	48	41 – 54	80	76 – 84	57	52 – 62	54	48 – 60

^a^Net survival estimated using general life tables

**Fig. 2:**
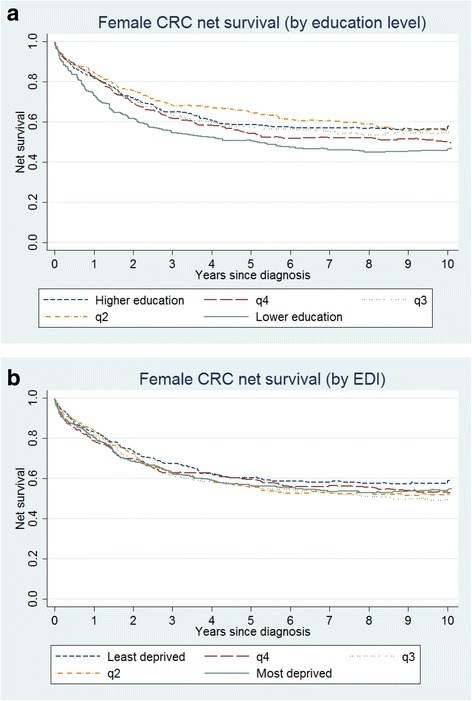
Net survival for female patients: **a** by education level and **b** by EDI group (general Life Tables)

By contrast, the pattern in survival across the five education levels was not gradual among women (Fig. 2a). Female patients coming from areas with the lowest education level presented always the lowest net survival over time. However, net survival hardly differed between the other education groups. Female net survival was also very similar between EDI groups, and not even the most deprived group detached from the remaining (Fig. 2b). Age-standardization of net survival estimates did not modify the survival pattern between education and EDI groups (Additional file 1: Table S1).

Adjusted excess hazard ratios (EHR) were computed from flexible parametric models with time-dependent effects for age and education and for age and EDI. We first used general life tables (i.e., not SES-specific). For male patients, the model confirmed the trend in increasing age-adjusted excess hazard across the education groups, more marked at longer term (Table 3). The excess hazard of death became significantly higher in the lowest educated group than in the highest educated (reference) group at 5 years (EHR = 1.40; 95 % CI: 1.06–1.84) and at 10 years (EHR = 1.51; 95 % CI: 1.08–2.11). For female patients, although the excess hazard in the lowest educated group was higher than the reference group, no statistically significant differences were found at 5 and 10 years since diagnosis (Table 3).

**Table 3:** Excess Hazard Ratio estimates (and 95 % Confidence Intervals) by education level and EDI (adjusted for age)^a^

		Male						Female					
		1-year		5-years		10-years		1-year		5-years		10-years	
		HR	95 % CI	HR	95 % CI	HR	95 % CI	EHR	95 % CI	EHR	95 % CI	EHR	95 % CI
Education level													
	Higher education	1		1		1		1		1		1	
	q4	1.10	0.88 – 1.36	1.16	0.89 – 1.50	1.18	0.86 – 1.62	1.04	0.83 – 1.32	1.21	0.92 – 1.59	1.29	0.90 – 1.83
	q3	1.15	0.92 – 1.43	1.27	0.97 – 1.65	1.32	0.95 – 1.82	1.02	0.81 – 1.30	1.05	0.78 – 1.41	1.06	0.73 – 1.55
	q2	1.13	0.90 – 1.42	1.27	0.97 – 1.67	1.34	0.96 – 1.87	0.84	0.65 – 1.09	0.88	0.64 – 1.21	0.90	0.60 – 1.35
	Lower education	1.16	0.92 – 1.46	1.40	1.06 – 1.84	1.51	1.08 – 2.11	1.33	1.03 – 1.71	1.27	0.93 – 1.75	1.25	0.83 – 1.87
EDI													
	Least deprived	1		1		1		1		1		1	
	q4	1.20	0.93 – 1.53	0.93	0.68 – 1.26	0.84	0.58 – 1.22	1.15	0.87 – 1.52	1.05	0.75 – 1.48	1.01	0.65 – 1.55
	q3	1.04	0.81 – 1.33	1.07	0.80 – 1.43	1.08	0.76 – 1.55	1.10	0.83 – 1.44	1.26	0.91 – 1.74	1.33	0.88 – 2.01
	q2	1.19	0.94 – 1.51	1.30	0.98 – 1.71	1.34	0.95 – 1.88	1.06	0.81 – 1.39	1.14	0.83 – 1.57	1.18	0.79 – 1.76
	Most deprived	1.14	0.90 – 1.43	1.25	0.96 – 1.64	1.30	0.93 – 1.82	1.15	0.89 – 1.48	1.02	0.75 – 1.40	0.97	0.65 – 1.45

^a^Excess hazard ratios estimated using general life tables

For male patients, the age-adjusted excess hazards for the more deprived groups were almost always higher than the one observed for the reference group (least deprived). However, the EDI-related pattern of changes in excess hazard ratios was not as clear as with education. Again, no clear association between EDI and excess hazard was found among women.

To evaluate the sensitivity of the results, the excess hazard ratios by education and EDI level were re-estimated using different sets of life tables.

**Fig. 3:**
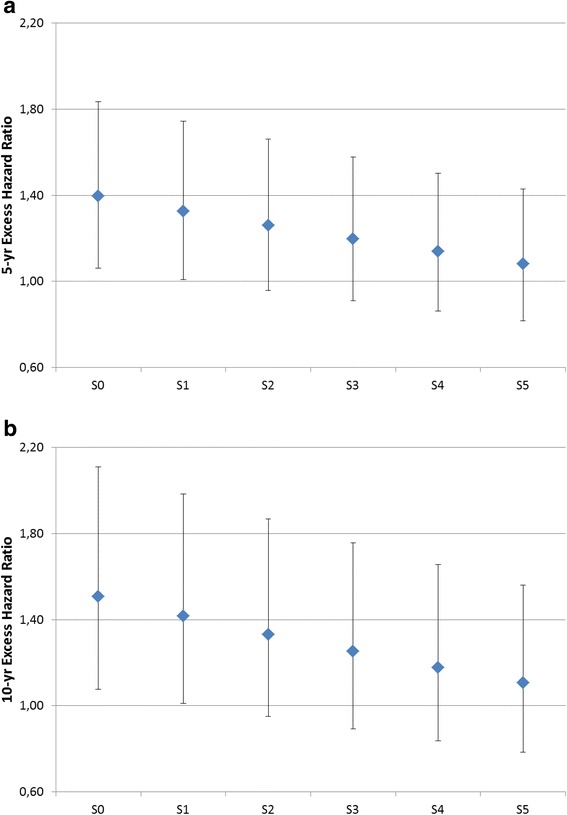
Sensitivity analysis – Excess Hazard Ratios for the least educated group (compared with most educated group) at **a** 5 years and **b** 10 years since diagnosis (male patients)

**Fig. 4:**
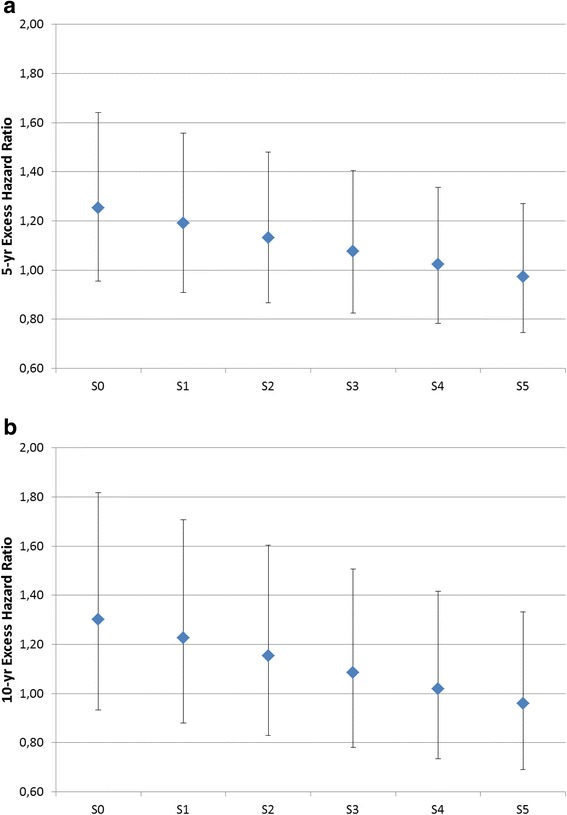
Sensitivity analysis – Excess Hazard Ratios for the most deprived group (compared with least deprived group) at **a** 5 years and **b** 10 years since diagnosis (male patients)

Overall, among men, the effect of education level variable was no longer significant in the excess hazard model as soon as fairly small inequalities in background mortality were considered (scenario S2). Figure 3 presents the excess hazard ratios at 5 (Fig. 3a) and 10 (Fig. 3b) years since diagnosis for the lowest education group, compared to the highest education group. Excess hazard at 5 and 10 years remained significantly different between the two extreme education groups only for narrow disparities in background mortality of the general population (scenarios S0 and S1). The excess hazard at 10 years of the least educated group was 51 % higher than the excess hazard of the group with highest education when using general life tables (S0). This difference reduced to 11 % when considering the English gap in background mortality (S5). For the EDI (Fig. 4a), the excess hazard ratio at 5 years between the most deprived group and the least deprived one reduced from 1.25 (S0) to less than one (S5). A similar behaviour was observed at 10 years (Fig. 4b).

Among women, as expected, the initial lack of inequalities observed with the general life tables remained for all scenarios (Additional file 2: Figures S1, S2).

## Discussion

When the expected (or background) mortality of the cancer patients was provided by general life tables, net survival from colorectal cancer tended to decrease with decreasing education level in men. These inequalities however occurred only for long-term survival, i.e., at 5 and 10 year since diagnosis. No clear gradient was observed for women, in spite of a general worse survival in the less educated group.

Inequalities in survival were in general smaller by EDI level than by education. This was true for both genders.

General life tables assume that the patients have the same (age-, sex- and calendar year-specific) expected mortality, regardless their education or EDI level, which is unlikely. It may result in an overestimation of the survival gap [30], in particular as time since diagnosis is increasing, as illustrated by our results. In the absence of education-specific or EDI-specific life tables in Portugal, we performed a sensitivity analysis, using hypothetical life tables adjusted for the respective SES measure. This analysis revealed that differences in expected mortality reduced considerably the observed inequalities in net survival. Fairly small education-related differences in expected mortality (scenario S2 – Fig. 3a) were sufficient to cancel the inequalities in net survival between education groups initially observed (S0). Scenario S2 corresponds to a difference in life expectancy as small as 3.1 years between the most educated and least educated categories in the general population, a difference which is likely to underestimate the real disparities in background mortality between socioeconomic or education groups in Portugal (i.e., still to overestimate the cancer survival gap). The gap in life expectancy in that scenario is for example smaller than the difference (3.6 years) observed between the North Region and the Portuguese islands (Madeira and Azores) [31], where the lowest life expectancy at birth in Portugal is observed. Disparities in background mortality are plausible since there is also strong evidence of worse health status in more deprived classes. Higher prevalence of cardiovascular disease, stroke, ischemic heart disease, hypertension, diabetes, obesity and low physical inactivity has been associated with lower socioeconomic status in Portugal [32]. In the Metropolitan Area of Porto, increased early mortality rates have been shown in more deprived parishes [33].

Although the general conclusions were similar, results obtained with education and EDI differed. The analysis of the area typology reveals that education level seems to be more related to a rural/urban distinction than EDI. While about 40 % of the patients coming from the least educated areas live in rural areas, only 13 % of the patients living in the more deprived areas correspond to rural zones. Since the major treatment centres are in urban areas, this suggests that the least educated patients have a worse accessibility to treatment centres. This is in accordance with Pinto et al. [19] that identified regional disparities in access to health care facilities as one of the major problems in the management of diagnosis and treatment of colorectal cancer patients.

Differential participation rate in screening programmes by socioeconomic condition is a source of inequalities in survival. In the region considered in this study however, no organized CRC screening programme existed during the period of diagnosis analysed, neither is yet implemented at the present. In Portugal, an official pilot CRC screening programme was initiated in 2009 in the centre region. In 2014, CRC screening programmes covered only 3.7 % of the Portuguese population [34]. Participation in opportunistic screening remained also low: a questionnaire study performed in Porto municipality in 2009 showed that about two thirds of the inquired (mean age 60 years-old) had never performed any type of CRC screening exam [35]. This study found no association between the knowledge of CRC risk factors and education level.

The association between CRC and socioeconomic factors has been evaluated in different countries with different health care systems [5, 6]. Some methodological differences in published studies can be pointed out. First, socioeconomic condition is defined either at individual level [7–9] or using an ecological measure [11, 12, 14]. Second, the metric to measure socioeconomic condition varies. Third, the outcome used is not homogeneous. Overall [36–38], cancer-specific [39, 40] or relative survival [7, 9, 41] have been used as outcome measures. Beyond these differences, most studies found an association between socioeconomic condition and survival from colorectal cancer.

A Danish study found a lower relative survival at 1 and 5 years for colon and rectum cancer patients with basic or high school, relatively to patients with vocational or higher education, for both genders [7]. Improved survival for more highly educated men was observed in Sweden for both colon and rectum cancers, compared with men with less than 9 years of completed education, while for women this difference was observed only for colon cancer [8]. Another study in Sweden found also a clear pattern of better survival for more highly educated groups [9]. Socioeconomic inequalities in colon and/or rectum cancer survival have also been found in England [11] and Japan [12]. Gorey and co-workers evaluated the association between income and colon cancer survival in San Francisco (US) and Toronto (Canada) [36]. Survival in San Francisco was significantly worse among people living in lower-income neighbourhoods. For Toronto though, no association was found between income and survival. Systemic health care issues, such as different health insurance coverage, were pointed out as the most plausible explanations for their findings. By contrast, still in the US, no evidence of racial (very much associated with SES in the US) inequalities were found within the Veterans Administration system in the US, a health care system with universal access [42]. Other studies found no association between socioeconomic condition and cancer outcome when comparing patients that had been offered treatment of the same type and same quality [43, 44]. In France, a small association was found between material deprivation and colorectal cancer survival [10]. However, the deprivation gap might have been overestimated since no deprivation-specific life tables were used. Other studies were inconclusive because they were based on overall survival or relative survival without deprivation-specific life tables [14, 37, 45]. Contrarily to these studies, we took in consideration the impact of plausible disparities in background mortality. The universal access nature of our healthcare system and the existence of a major public cancer reference centre which treats an important proportion of cancer patients of the north region could help explain the lack of association found between SES and survival. Nevertheless, further studies are needed to better understand between countries differences in the patients’ pathway and healthcare organization that explain the existence or not of cancer survival inequalities.

Net survival was estimated in this study using the recently proposed estimator by Pohar-Perme [24]. This is an unbiased non-parametric estimator of the quantity of interest [46], when high quality information on cause of death is not available. Cancer data were provided by a population-based cancer registry (RORENO) that has been shown to have high completeness [47].

This study has some limitations that should be pointed out. We used area-based variables due to the absence of individual information. This can lead to some dilution of the effect. The education and the EDI levels attributed to each patient represent though the environment of his/her residence and not necessarily the individual condition. Furthermore, many other studies on the association between SES and survival from cancer have used ecological socioeconomic indices and still were able to find significant associations [11, 12, 14]. It has been shown that the size of the geographic unit is a key element for detecting inequalities [13]. The geographic unit we used to attribute the education level to each patient had a median population of 660 inhabitants, which correspond to a size comparable or lower than what has been used in those other similar studies. Another limitation of the study is the lack of information on stage of disease at diagnosis. Also information on comorbidities and treatment was not available.

Education level was measured as the proportion of individuals with at least nine years of education, i.e., the compulsory level of education in Portugal until recently. We have also used four years of education as cut-off, since this was the former compulsory level of education, and the results were similar (data not shown).

Patients analysed in this study were diagnosed in the period 2000-2002 which allowed for a long-term follow-up. These years correspond though to a period well before the economic crisis that began in 2008 and which affected Europe and particularly south European countries including Portugal. The National Health Service has been subject in recent years to budgetary constraints which may have led to inequalities in access to healthcare. Evaluations similar to the one presented in this study should be performed in the near future to access the impact of recent health policies in cancer survival inequalities. Other cancer sites should be analysed also to confirm, or not, the findings in this study.

The EDI is a recently developed indicator of socioeconomic deprivation. For Portugal the main variables used in the construction of this index were overcrowding, no indoor flushing, education, unemployment and not owning a house, reflecting though different domains of deprivation. Our study is one of the first studies to use this index. It would be interesting to compare SES inequalities in cancer survival across countries using this same index.

## Conclusions

To the best of our knowledge, this is the first population-based study to address the question of socioeconomic inequalities in survival from colorectal cancer in Portugal. We found some inequalities in net survival by education level, but less by EDI, when using general life tables. However, the sensitivity analysis performed showed that these inequalities in cancer survival were most likely absent and were better explained by differences in background mortality. Our study confirms the importance of using the relevant life tables, or of performing sensitivity analysis, when evaluating socioeconomic inequalities in cancer survival.

## Abbreviations

AIC, Akaike information criteria; CI, confidence interval; CRC, colorectal cancer; EDI, European deprivation index; EHR, excess hazard ratio; ICD, international classification of diseases; RORENO, North Region Cancer Registry of Portugal; SES, socioeconomic status; SNS, National Health Service (“Serviço Nacional de Saude”)

## Additional files

Additional file 1: Table S1. Age-standardized net survival estimates by education level and EDI. (DOCX 18 kb) Additional file 2: Figure S1-S2. Figure S1 - Sensitivity analysis Education (Female Patients): Excess Hazard Ratios for the least educated group (compared with most educated group) at a) 5 years and b) 10 years since diagnosis. Figure S2 – Sensitivity analysis EDI (Female Patients): Excess Hazard Ratios for the most deprived group (compared with least deprived group) at a) 5 years and b) 10 years since diagnosis. (DOCX 92 kb)
