# Supplementary material for: No inequalities in survival from colorectal cancer by education and socioeconomic deprivation - a population-based study in the North Region of Portugal, 2000-2002
Source: BMC Cancer. 2016 Aug 5;16:608. doi: 10.1186/s12885-016-2639-9 (PMC4975888; doi:10.1186/s12885-016-2639-9)
Supplement: Additional file 1: Table S1. — Age-standardized net survival estimates by education level and EDI. (DOCX 18 kb) [file 12885_2016_2639_MOESM1_ESM.docx]

**Table S1 – Age-standardized net survival estimates by education level and EDI**

|  |  | **Male** | | | | | | | |  | **Female** | | | | | | | |
| --- | --- | --- | --- | --- | --- | --- | --- | --- | --- | --- | --- | --- | --- | --- | --- | --- | --- | --- |
|  |  | **1-year** | |  | **5-years** | |  | **10-years** | |  | **1-year** | |  | **5-years** | |  | **10-years** | |
|  |  | **%** | **95% CI** |  | **%** | **95% CI** |  | **%** | **95% CI** |  | **%** | **95% CI** |  | **%** | **95% CI** |  | **%** | **95% CI** |
| Education level | |  |  |  |  |  |  |  |  |  |  |  |  |  |  |  |  |  |
|  | Higher education | 80 | 76 - 84 |  | 59 | 54 - 64 |  | 55 | 48 - 63 |  | 81 | 77 - 85 |  | 58 | 53 - 63 |  | 55 | 49 - 61 |
|  | q4 | 79 | 75 - 82 |  | 58 | 52 - 63 |  | 51 | 44 - 58 |  | 82 | 79 - 86 |  | 53 | 48 - 58 |  | 49 | 43 - 55 |
|  | q3 | 83 | 79 - 86 |  | 56 | 50 - 61 |  | 48 | 41 - 55 |  | 82 | 78 - 86 |  | 57 | 51 - 63 |  | 54 | 47 - 61 |
|  | q2 | 82 | 77 - 86 |  | 53 | 47 - 58 |  | 42 | 36 - 48 |  | 85 | 81 - 89 |  | 65 | 59 - 71 |  | 55 | 48 - 63 |
|  | Lower education | 82 | 78 - 86 |  | 54 | 48 - 60 |  | 42 | 36 - 49 |  | 74 | 68 - 79 |  | 51 | 44 - 58 |  | 46 | 38 - 53 |
|  |  |  |  |  |  |  |  |  |  |  |  |  |  |  |  |  |  |  |
| EDI | |  |  |  |  |  |  |  |  |  |  |  |  |  |  |  |  |  |
|  | Least deprived | 80 | 76 - 85 |  | 58 | 52 - 64 |  | 52 | 44 - 60 |  | 82 | 78 - 87 |  | 59 | 52 - 65 |  | 55 | 48 - 63 |
|  | q4 | 80 | 75 - 84 |  | 56 | 51 - 62 |  | 57 | 48 - 65 |  | 78 | 73 - 83 |  | 59 | 53 - 65 |  | 52 | 45 - 59 |
|  | q3 | 81 | 77 - 85 |  | 58 | 52 - 63 |  | 45 | 39 - 51 |  | 82 | 77 - 86 |  | 55 | 49 - 61 |  | 48 | 41 - 55 |
|  | q2 | 79 | 75 - 83 |  | 54 | 49 - 59 |  | 44 | 37 - 51 |  | 84 | 80 - 88 |  | 56 | 50 - 61 |  | 52 | 45 - 58 |
|  | Most deprived | 83 | 80 - 86 |  | 55 | 50 - 59 |  | 48 | 41 - 54 |  | 80 | 77 - 84 |  | 56 | 51 - 61 |  | 53 | 47 - 59 |
