# Supplementary material for: No inequalities in survival from colorectal cancer by education and socioeconomic deprivation - a population-based study in the North Region of Portugal, 2000-2002
Source: BMC Cancer. 2016 Aug 5;16:608. doi: 10.1186/s12885-016-2639-9 (PMC4975888; doi:10.1186/s12885-016-2639-9)
Supplement: Additional file 2: Figure S1-S2. — Figure S1 - Sensitivity analysis Education (Female Patients): Excess Hazard Ratios for the least educated group (compared with most educated group) at a) 5 years and b) 10 years since diagnosis. Figure S2 – Sensitivity analysis EDI (Female Patients): Excess Hazard Ratios for the most deprived group (compared with least deprived group) at a) 5 years and b) 10 years since diagnosis. (DOCX 92 kb) [file 12885_2016_2639_MOESM2_ESM.docx]

a)
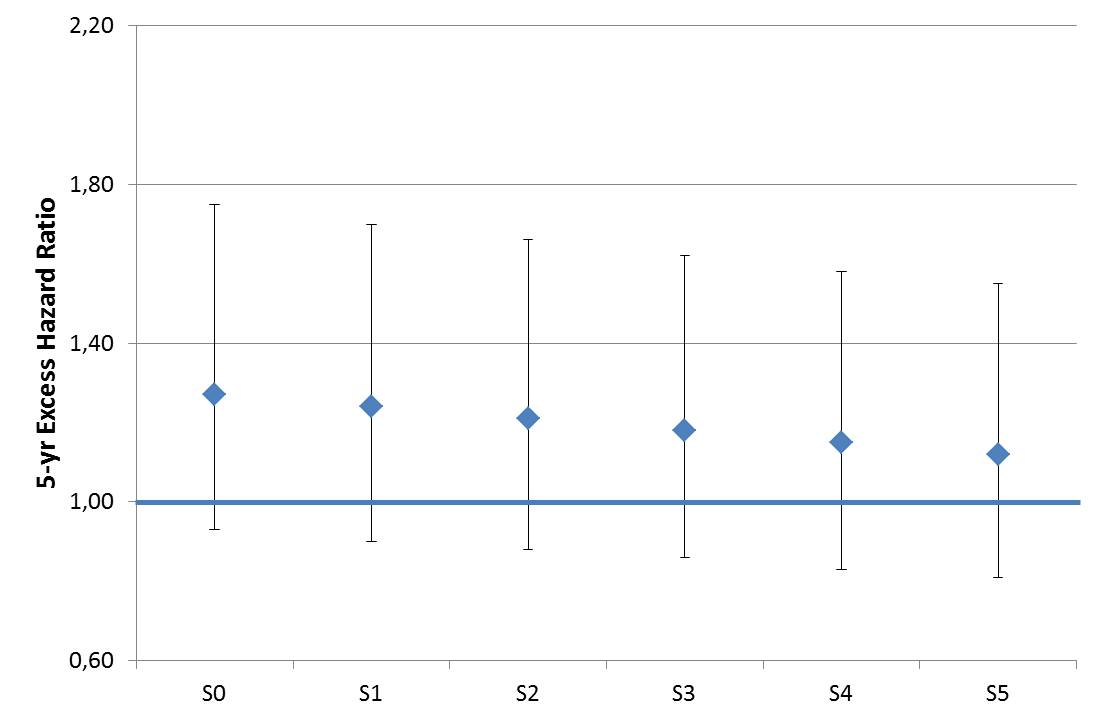
 b)
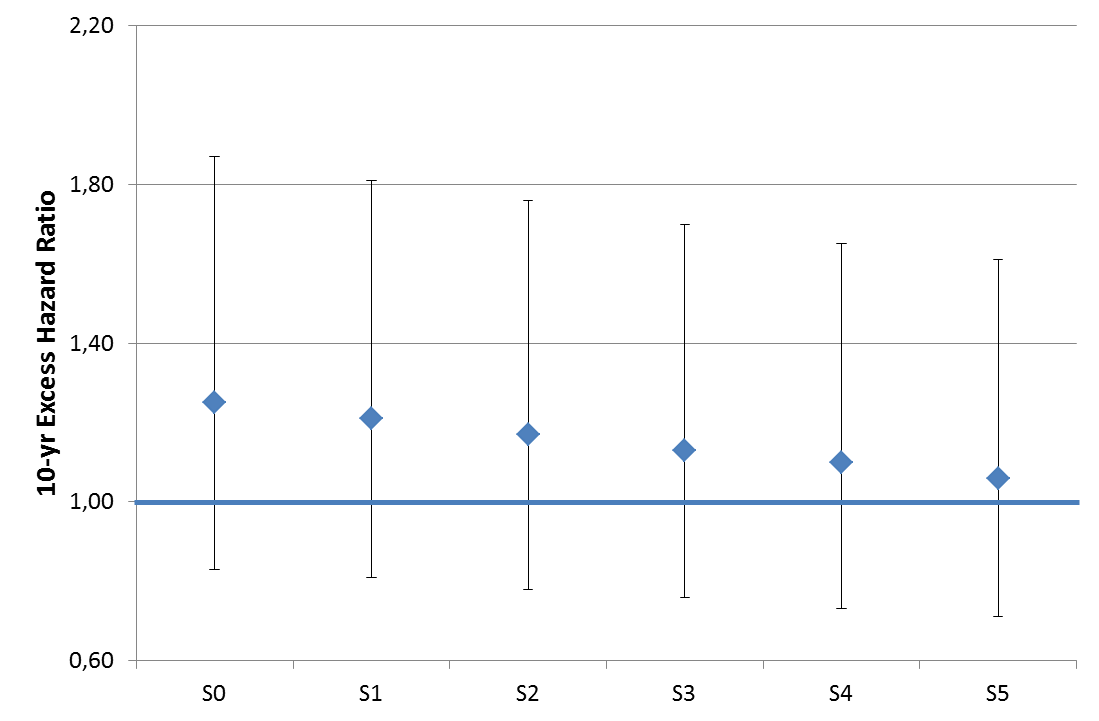


**Figure S1 – Sensitivity analysis Education (Female Patients): Excess Hazard Ratios for the least educated group (compared with most educated group)**

**at a) 5 years and b) 10 years since diagnosis.**

S0 – general life tables

S1 – education-specific life tables with 20% of the English gap between education groups

S2 - education-specific life tables with 40% of the English gap between education groups

S3 - education-specific life tables with 60% of the English gap between education groups

S4 - education-specific life tables with 80% of the English gap between education groups

S5 - education-specific life tables with the English gap between education groups

a)
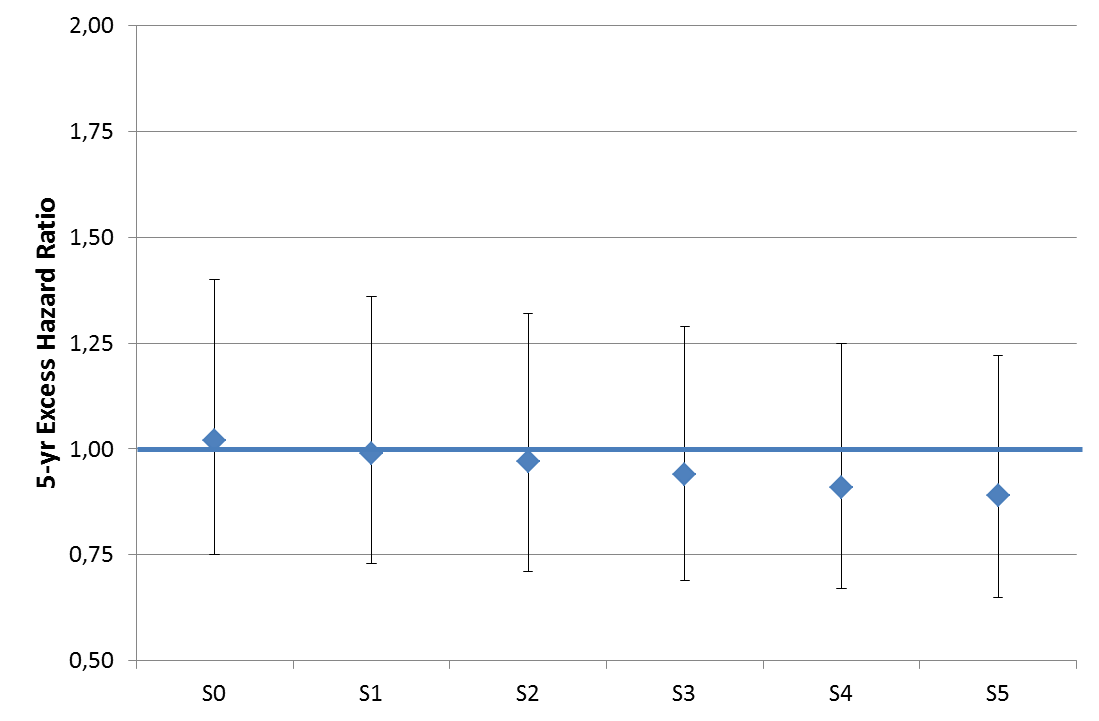
 b)
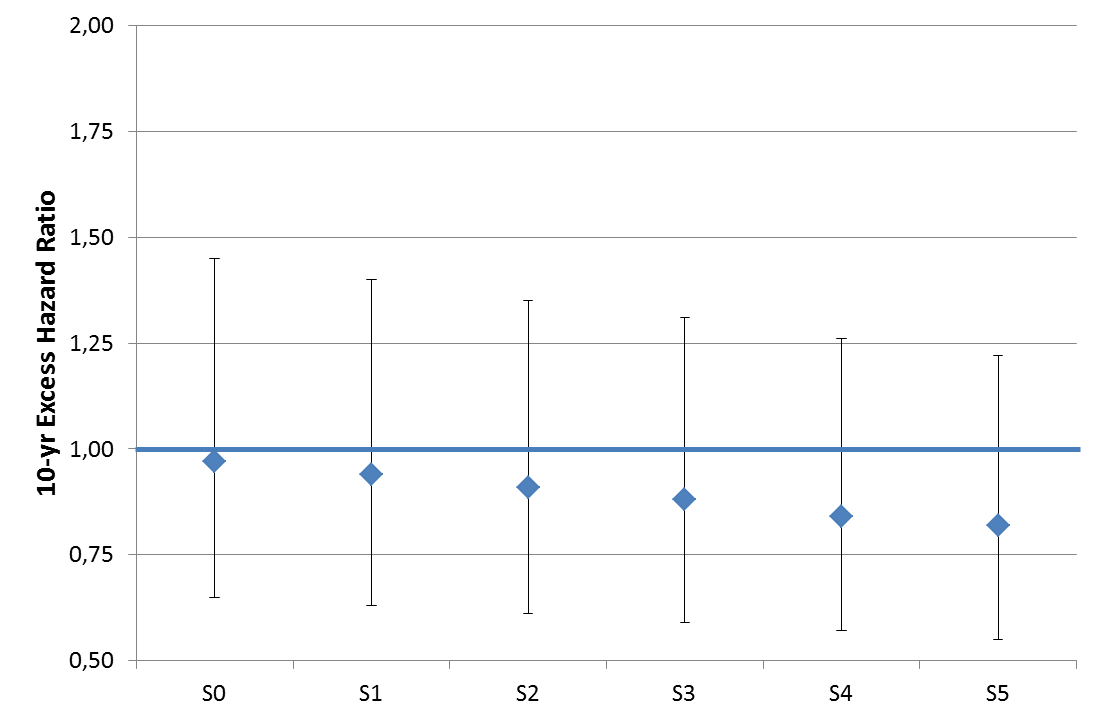


**Figure S2 – Sensitivity analysis EDI (Female Patients): Excess Hazard Ratios for the most deprived group (compared with least deprived group)**

**at a) 5 years and b) 10 years since diagnosis.**

S0 – general life tables

S1 – EDI-specific life tables with 20% of the English gap between deprivation groups

S2 - EDI-specific life tables with 40% of the English gap between deprivation groups

S3 - EDI-specific life tables with 60% of the English gap between deprivation groups

S4 - EDI-specific life tables with 80% of the English gap between deprivation groups

S5 - EDI-specific life tables with the English gap between deprivation groups
